# From Residential Care to Supportive Housing for People With Psychiatric Disabilities: Past, Present, and Future

**DOI:** 10.3389/fpsyt.2019.00862

**Published:** 2019-11-22

**Authors:** Marianne Farkas, Steve Coe

**Affiliations:** ^1^Center for Psychiatric Rehabilitation, Sargent College, Boston University, Boston, MA, United States; ^2^Community Access, New York, NY, United States

**Keywords:** supported housing, serious mental illnesses, recovery, psychiatric rehabilitation, supportive housing, implementation challenges

## Abstract

For centuries, treatment and accommodation for people with significant mental health conditions in many countries, including the United States, have been viewed as necessarily inseparable elements, first in asylums and then, with deinstitutionalization, in community care models. The advent of psychiatric rehabilitation and later, recovery, helped to shift the paradigm of mental health services and the role of housing, to one focused on promoting the ability of individuals to achieve not only a life *located in* the community, but one that reflects a meaningful life as *part* of a community. In this context, supportive housing emerged as a model based on integrated, permanent, affordable housing, selected by the person, with flexible supports that are functionally separate, but available as needed and wanted. This model of housing has been predominant in American mental health services for over 20 years, and evidence now exists for its outcomes in terms of housing stability, symptom reduction, and psychosocial variables. Current challenges, both at the societal and the individual level, confront the sustainability of supportive housing, with some efforts being made by housing groups to address these challenges. This article reviews the evolution of supportive housing and its basic tenets, identifying the challenges and some efforts to address them. In addition, the article discusses the current social and economic climate, which appears to be shaping opposing trends, and makes a call to action, to mitigate the possible risks to the future of this value-based housing approach.

## Introduction

Access to shelter is a right enshrined in the International Convention on Human Rights (1). Beyond being a basic right, housing contributes to a sense of identity and community for most people (2, 3). For at least the past 200 years, mental health and rehabilitation treatment providers, advocates, and government entities alike have struggled to provide places to live for people with significant mental health conditions. Adults with significant mental health conditions^1^have often encountered barriers ranging from, but not limited to, discrimination, poverty, a paucity of available housing, lack of supports oriented to their recovery and social isolation when trying to succeed in living with family or in another residence.

This article presents the experience of the American response to the question of housing for people with significant mental health conditions, to highlight the need for continued vigilance and efforts to expand and sustain supportive housing, in the face of current challenges and potential retrenchment.

### Evolution of the US Perspective

The major historical milestones underpinning our current beliefs about community living for people with psychiatric disabilities include the establishment of hospitals, deinstitutionalization, the development of community care, and the emergence of rehabilitation and recovery approach to services.

#### Hospitals as Housing

As occurred in most high income countries, the 18th and 19th Century American response to dealing with significant mental health conditions, was to segregate the population, whether in almshouses, as was done in Colonial America, or in “asylums” (4). Both in France and in America, the idea of “moral treatment” emerged during the Age of Enlightenment. In France, the psychiatrist Pinel established asylums to cure mental illness using this approach. In America, the Quakers or “the Society of Friends,” similarly established the first private psychiatric hospital, i.e., “The Friends' Asylum for the Relief of Persons Deprived of the Use of Their Reason,” in 1813. Their basic religious tenets included the belief that God dwelt in every person, and therefore, all individuals should be treated equally and with respect. Moral treatment included everything from exercise and religious training, to lessons on good hygiene and activities tailored to each person's interests, such as writing or music (4, 5), and was based on the principle of creating a therapeutic environment in which housing and compassionate support was integrated in one place. Unfortunately, most of these ideals did not endure long. Over the following century, these small rural retreats evolved into over-crowded, publicly-operated institutions with their function transitioning from the promotion of healthy living, to one of reducing perceived community risk, through intense supervision (6).

#### Deinstitutionalization and Community Care

By the 1950s, there were approximately 560,000 individuals living in psychiatric hospitals in the United States (5). Public exposure to the deplorable conditions in state hospitals in the 1950s, the rise of new psychotropic medications, and the emergence of various civil rights movements in the 1960s led to a demand for the transfer of long stay inpatients to the community, as was eventually policy in most high-income countries (7). In the United States, the inpatient census fell approximately 76% by the 1980s, with 130,000 people remaining as inpatients at that time (8).

As state hospital use was reduced in favor of community care, the issue of where people with significant mental health conditions would reside gained prominence. Negative neighborhood reactions in response to publicized increases in homelessness, was also associated with deinstitutionalization in other countries to varying extent (9, 10). The earliest housing models developed in response to deinstitutionalization, retained a belief in integrating accommodations and treatment. These residential care and treatment models were usually highly structured, long-term care facilities, such as group homes (i.e., multiple residents living in a structured environment with 24 hour supervision); board-and-care homes (homeowners paid to provide food and lodging for one or more individuals) and halfway houses (i.e., group homes intended to be an interim residence between inpatient hospitalization and more independent living). Residents were expected to follow house rules designed to promote transition to less intensive services. Gradually, a “linear” residential model or a “continuum of care” system developed in the late 1970s and 1980s, in which a person was to progress from the hospital, through halfway houses, group homes, and finally, supervised apartments (11, 12).

#### Rehabilitation and Recovery

It became clear, however, that many such residential care and treatment homes did not, in fact, help individuals gain control over their own lives. A seminal article (13), asked the question “Halfway homes: halfway to where?”. It noted that, contrary to the original intent of moving people out of institutions, smaller versions of highly supervised, regulated, and to a large extent, segregated residential environments trapped residents in a kind of trans-institutionalization, a development also seen in European countries (14). Similar research on the shortcomings of this model led to a move away from a linear residential model to the emergence of the U.S. Federal Community Support System (CSS), which identified the need for more than just physical housing or symptom reduction services for people to achieve true community integration (15). The CSS made explicit that services needed to support people with significant mental health conditions' societal goals, such as jobs, school, a life partner, and health. The model mandated 10 distinct services to achieve these objectives, e.g., dental care, rehabilitation, treatment, case management, etc.

Psychiatric rehabilitation, using a biopsychosocial/social disability model (16) emphasized an ecological approach or a “person-environment fit.” It evolved as a service to help people develop the skills and supports they needed for the kind of goals they themselves wanted. Psychiatric rehabilitation also made choice a central feature of its process and provided structured interventions to help individuals make their aspirations and choices a reality (17–21). Housing goals were seen as a matter of preference rather than a function of performance level, or category of illness. Services began to be separated from residences, focusing on helping people develop the ability to achieve these goals. The radical concept that a “home is just a home” promoted “real world” places and activities (e.g. sports arenas, banks, public buses), as more appropriate venues for skill development, in order for people to gain greater independence (12, 22).

First-person accounts (e.g. (23) and longitudinal studies (e.g. (24) led to the acknowledgment of the possibility of recovery or achieving a meaningful life, despite symptoms or illness (e.g. (25, 26), an idea gradually accepted internationally (26). In the United States, recovery emerged as a vision for services in the 1990s, strengthening the importance of including individuals with “lived experience” of mental illnesses in designing, delivering, and evaluating services, paving the way for the development of a peer workforce (27, 28). The 1980s humanitarian idea of housing as more than a location for treatment (e.g. (29), was eventually confirmed. Research began to suggest that it was a critical pathway for recovery by providing a sense of “place” for “being, doing, becoming, and belonging” in a community (30).

Earlier beliefs in the importance of a residential continuum required people to move based on functioning, but were now understood to result in lost relationships and fragmentation of communities (17) and thus counter-productive to people's recovery. The recognition that housing was a key factor in promoting social inclusion and citizenship (31, 32) led to housing models that incorporated these aspects of daily life. As a result, permanent, affordable housing paired with flexible, user-driven supports is now the prevailing model of high-quality permanent supportive housing (PSH) in the United States.

### What Is Supportive Housing in the United States?

Studying mental health-supported accommodations is hampered around the world by a confusion of terms^2^and characteristics, such as models, physical structures, and recovery focus (33, 34). A recent effort to create a taxonomy identified at least five different international types varying along dimensions of staffing location, level of support, permanence, physical setting (35). The US PSH models have two essential components: 1) housing is permanent, not transitional; 2) supportive services are not required of the tenant to live there (36). Supportive housing typically mirrors the tenant rules and expectations of any standard housing type, in which leases and standard house rules define the expectation of both the tenant and landlord. Housing itself is seen as the platform for personal growth and having a stable home directly impacts one's physical and mental health (37, 38). To enhance community integration, PSH providers have added housing features such as computer centers, urban farms, and exercise rooms, along with services, such as case management, peer support, and others (30).

The basic principles of US supportive housing include (39):

- The individual owns the housing/has a lease in his or her own name;
- Housing is integrated into the community;
- Housing is affordable (i.e. no more than 40% of adjusted gross income);
- Services offered are not a condition for tenancy.

The most frequently studied PSH supportive housing program model is “Housing First,” originally designed for homeless individuals with the most complex behavioral health conditions. Housing First provides individuals with immediate access to housing, regardless of their functioning or use of substances; client choice is emphasized in every aspect of treatment, with a harm reduction approach to substance abuse (40). Along with solid evidence for housing retention and stability and appropriate use of clinical services over time, there is some, albeit inconsistent evidence, that this approach is also associated with improvement in symptoms, quality of life, and social functioning [e.g. (41)]. An international systematic review of supportive housing indicates that people who have moved out of long-term psychiatric hospitals to such housing programs, demonstrate improvement or non-deterioration in psychiatric symptoms, social functioning, and reduced rates of rehospitalization (42). Supportive housing outcomes for individuals of the “post deinstitutionalization era” (i.e., those who never had extended hospital stays) are more mixed due to the complexity of designing these studies and the few absolute numbers of these studies to date.

### Challenges in Supportive Housing

Access to safe, secure housing has been acknowledged as a critical element in the recovery process for more than 20 years. The practicalities of building and managing housing with recovery-oriented services, however, have presented serious challenges in sustaining and expanding its availability. Challenges include staff capacity to deliver recovery-oriented support, housing affordability, and the effects of race and discrimination among others.

#### Staff Capacity to Promote Recovery

A major challenge to implementing PSH has been the providers' ability to shift paradigms of service from control, risk reduction, and chronic illness, to the foundational elements of supporting choices, the development of new skills, health, and wellness. Navigating the boundaries of staff input versus personal choice for example, has always been difficult in mental health services (43), but especially in supportive housing programs where the emphasis is on maintaining a home, rather than a treatment setting. Vestiges of the historical institutional framework are still apparent in many supportive housing programs, such as restrictions on visitation in the homes, requirements for medication oversight that mix treatment services with the housing service, the inclusion of service staff in landlord-tenant relationships, or the segregation of residents by including only people with disabilities as tenants. Further complicating this problem is the lack of sustainable funding to attract qualified people and provide them with ongoing training, or advancement opportunities to retain experienced staff. Without the consistent capacity to provide recovery-oriented support services, PSH can easily become a locus for mini-institutions in the community, instead of homes for people who are part of their communities.

#### Housing Affordability

Permanent supportive housing also needs an affordable housing stock to draw from. During the early deinstitutionalization period, ex-patients had access to government income support that often provided enough to rent rooms in the housing market of the 1970s, albeit usually in the least desirable units available. Eventually, however, even these low cost housing resources were lost to the effects of gentrification and urban renewal. In New York City alone, it is estimated that over 100,000 low cost units were lost during the 1980s (44).

The lack of affordable housing is an ongoing growing issue that affects the American general population. The average cost of housing in the most expensive cities (e.g. Los Angeles, New York, Boston), has increased by 224% since 2000 (45), with a significant increase in the number of renters paying more than 50% of their income (46, 47). Increased costs and lack of income growth push already vulnerable and marginalized people, like those with psychiatric disabilities, into shelters and homeless encampments.

#### Discrimination and Race

Prejudice against individuals with psychiatric disabilities, diminishes their social capital, adding even tighter limits on the kinds of housing choices people can make. Despite laws against housing discrimination, landlords or neighborhoods are often resistant to people in mental health recovery as residents, reducing an already small pool of available options (48–50). The discrimination affects African Americans disproportionately, so that they comprise approximately 40% of homeless individuals, even though this population represents only 12.5% nationally (51). It is estimated that up to 50% of those who are homeless both in Western Europe and North America, have significant mental health issues (52).

## Discussion

The American experience may provide an optimistic, albeit cautionary tale about sustaining supportive housing. On the one hand, it is a well-established service in the array of U.S. mental health service systems, with documented outcomes for homeless and deinstitutionalized populations, as well as moderate but growing evidence of effectiveness for other groups with mental health conditions (53).

A systematic review of the international literature has suggested that supportive housing programs with a high degree of tenant satisfaction and stability have empathic staff who are expert in those psychiatric rehabilitation techniques which support informed choice and community participation (54). Such training in psychiatric rehabilitation techniques is now readily accessible through membership groups, such as Psychiatric Rehabilitation Association (https://www.psychrehabassociation.org), or training entities, such as the Center for Psychiatric Rehabilitation (https://cpr.bu.edu/develop) among others. These focus on teaching providers and supervisors how to support informed choice while engaging the person's own expertise to learn to live as members of the community.

Even the most highly trained and experienced staff can encounter difficult situations when supporting people with complex needs. Turnover among experienced staff may leave an organization with newer, less trained staff. Organizations have to be structured to respond in ways consistent with core recovery values (i.e., services that are person centered, in full partnership with peers, based on choice and hopefulness (27)), even when staff cannot. Cross disciplinary tools to promote recovery oriented services are available (e.g. Recovery Promoting Competencies' Toolkit, www.cpr.bu.edu/develop), as well as discipline specific tools (e.g. SAMHSA's Recovery to Practice curricula, https://www.samhsa.gov/recovery-to-practice/rtp-curricula). These address not only workforce development needs, but also provide tools that can support the fundamental reimagining of an organization's culture including mission, values, and personnel practices often required to deliver such services (23, 55, 56).

Efforts to expand options by overcoming the challenges of housing affordability for people with psychiatric disabilities have been underway in some states. Early advocacy efforts in New York, for example, helped to establish a “right to shelter” for homeless people as a government obligation. The obligation resulted in guaranteed financing that produced more than 40,000 supportive housing units since the 1990s (www.shnny.org). More recently, California created a special task force to address the state's homelessness epidemic and pledged to finance 3.5 million new housing units by 2025. These examples point to a growing understanding of the need for long-range planning and sustained commitment by government to finance the building and maintenance of supportive housing.

Another approach seeks to maximize participant choice in finding housing, by providing individuals with an annual housing and services allotment that they can spend anywhere within a proscribed set of guidelines. Known variously as “self-directed care” or “self-directed services,” research findings suggest that it has superior client outcomes and greater satisfaction with mental health care, compared to services as usual (57).

On the other hand, it is difficult to be as optimistic about the U.S. capacity to address ongoing discrimination. Issues such as gun violence and domestic terrorism have created a climate in which even the U.S. President (41) erroneously identifies people with mental illnesses as the cause (e.g. (58, 59). Fear-based responses to social issues have led to initiatives seeking greater societal control over choices made by people with significant mental health conditions that threaten the basic precepts of supportive housing (e.g. (60). This has rekindled old debates about the balance between reducing perceived risks to society and personal civil liberties.

The array of challenges to PSH are currently counter-balanced by efforts to increase the number of housing units, the growing variety of training, and tools to deliver recovery-oriented supports and funding innovations, such as self-directed care. Advocates and researchers alike, however, need to continue developing the case for PSH based in a recovery orientation as an essential component of the healthcare system. Access to permanent housing that is a home rather than a housing facility, must be expanded. Otherwise, people with significant mental health conditions are in danger of continuing to be overrepresented in our jails, shelters, and emergency rooms and living segregated lives in, not *of* their communities. Future sustainment requires both advocacy and more nuanced research to clearly identify and embed those features of supportive housing that produce the most tangible improvements in a person's well-being and, by extension, the economic and social value that well-being can bring to the community as a whole.

## Author Contributions

Both authors generated the important ideas included in the outline. SC contributed particularly to the *Challenges* section, as well as reviewing and editing draft manuscripts. MF wrote the majority of the article and finalized the manuscript.

## Funding

The writing of this article was partially supported by US National Institute on Disability, Independent Living and Rehabilitation Research within the Administration for Community Living; the Substance Abuse and Mental Health Services Administration, Center for Mental Health Services and the Department of Health and Human Services, Grant #90RT5029. The contents of this paper do not necessarily represent the policy of the above entities.

## Conflict of Interest

The authors declare that the research was conducted in the absence of any commercial or financial relationships that could be construed as a potential conflict of interest.

## Footnotes

This article uses “people first” language, with terms such as “people with significant mental health conditions,” “people in mental health recovery,” “people with psychiatric disabilities” interchangeably, to refer to individuals who live with challenges often described as “serious mental illnesses” of two or more years duration, and psychiatric symptoms that impair living, learning or working in a valued societal role Common diagnoses associated with people in this group include: schizophrenia, bipolar illnesses, and other psychotic illnesses; chronic depression etc.

Terms, such as “floating outreach,” “supported accommodation,” “supportive housing,” “housing with supports,” have all been used in international literature almost interchangeably. We use the term “permanent supportive housing” (PSH) to mean a specific model of the general category as described in the text.

## References

[1] United Nations Habitat The Right to Adequate Housing: Fact Sheet 21(Rev 1) Vol. 21 Geneva: UN Office of the High Commissioner for Human Rights (OHCHR) (2009). Available at: https://www.refworld.org/docid/479477400.html ISSN 1014-5567

[2] Chesters J Fletcher M Jones R Mental illness, recovery and place. Aust e-journal Adv Ment Heal (2005) 4(2):89–97. 10.5172/jamh.4.2.89

[3] Manzo LC Perkins DD Finding common ground: the importance of place attachment to community participation and planning. J Plan Lit (2006) 20(4):335–50. 10.1177/0885412205286160

[4] Benjamin LT Baker DBClinical psychology: mental asylum. In: From Séance to Science: A History of the Profession of Psychology in America. Wadsworth/Thomson Learning. (2014). p. 32–8.

[5] Charland LMoral treatment in the eighteenth and nineteenth centuries. In: Rudnick A Roe D. editors. Serious mental illness: Person-centered approaches [Internet]. Milton Key (2011). p. 19–25. Available from: https://trove.nla.gov.au/work/159134686?q&sort=holdings+desc&_=1571240396622&versionId=263971013.

[6] Bachrach LL Deinstitutionalization: An Analytical Review and Sociological Perspective. Superintendent of Documents, US Government Printing Office, Washington DC: DHEW publication (1976). 43 p.

[7] Thornicroft G Deb T Henderson C Community mental health care worldwide: current status and further developments. World Psychiatry (2016) 15(3):276–86. 10.1002/wps.20349 PMC5032514 27717265

[8] Lamb HR Weinberger LE The shift of psychiatric inpatient care from hospitals to jails and prisons. J Am Acad Psychiatry Law. (2005) 33(4):529–34. 16394231

[9] Goldman HH Morrissey JP The alchemy of mental health policy: homelessness and the fourth cycle of reform. Am J Public Health (1985) 75(7):727–31. 10.2105/AJPH.75.7.727 PMC1646312 4003648

[10] Fakhoury W Priebe S The process of deinstitutionalization: an international overview. Curr Opin Psychiatry [Internet]. (2002) 15(2):187–92. 10.1097/00001504-200203000-00011 Available from: https://journals.lww.com/co-psychiatry/Fulltext/2002/03000/The_process_of_deinstitutionalization:an.11.aspx.

[11] Barrow S Zimmer R Transitional Housing and Services: a Synthesis. In: Fosburg L Dennis D. editors. Practical Lesson. 1998 Symposium on Homelessness Research (1999). p. 10–1.

[12] Rog DJ The Evidence on Supported Housing. Psychiatr Rehabil J (2004) 27(4):334–44. 10.2975/27.2004.334.344 15222146

[13] Cometa MS Morrison JK Ziskoven M Halfway to where? A critique of research on psychiatric halfway houses. J Community Psychol (1979) 7(1):23–7. 10.1002/1520-6629(197901)7:1<23::AID-JCOP2290070105>3.0.CO;2-N 10240150

[14] Priebe S Frottier P Gaddini A Kilian R Lauber C Martínez-Leal R Mental health care institutions in nine European countries, 2002 to 2006. Psychiatr Serv (2008) 59(5):570–3. 10.1176/ps.2008.59.5.570 18451020

[15] Turner JC TenHoor WJ The NIMH community support program: pilot approach to a needed social reform. Schizophr Bull (1978) 4(3):319–48. 10.1093/schbul/4.3.319

[16] World Health Organization The International Classification of Impairments, Disabilities, and Handicaps (ICIDH): a manual of classification relating to the consequences of disease. Switzerland, Geneva (1980). 207p.

[17] Anthony WA Cohen MR Farkas MD Gagne C Psychiatric rehabilitation. Boston, MA: Center for Psychiatric Rehabilitation; (2002) p. 1–432.

[18] Anthony WA Farkas MD The essential guide to psychiatric rehabilitation practice. Boston, MA:Boston University Center for Psychiatric Rehabilitation; (2011) p. 1–73 p.

[19] Farkas M Anthony W Montenegro R Gayvoronskaya E Person Centered Psychiatry in Person-centered psychiatric rehabilitation. Switzerland: Springer International Pubs (2016), 277–89. 10.1007/978-3-319-39724-5_21

[20] Shern D Tsemberis S Anthony W Lovell A Richmond L Felton C Serving street-dwelling individuals with psychiatric disabilities. Am J Public Health (2000) 90(12):1873–8. 10.2105/AJPH.90.12.1873 PMC1446423 11111259

[21] Swildens W Van Busschbach JT Michon H Kroon H Koeter MWJ Wiersma D Effectively working on rehabilitation goals: 24-month outcome of a randomized controlled trial of the Boston psychiatric rehabilitation approach. Can J Psychiatry (2011) 56(12):751–60. 10.1177/070674371105601207 22152644

[22] Farkas M Introduction to psychiatric/psychosocial rehabilitation (PSR): History and foundations. Curr Psychiatry Rev (2013) 9(3):177–87. 10.2174/1573400511309030003

[23] Onken S Craig CM Ridgway P Ralph RO Cook J An analysis of definitions and elements of recovery: a review of the literature. Psychiatr Rehabil J (2007) 31(1):9–22. 10.2975/31.1.2007.9.22 17694711

[24] Harding CM Zahniser JH Empirical correction of seven myths about schizophrenia with implications for treatment. Acta Psychiatr Scand (1994) 90(s384):140–6. 10.1111/j.1600-0447.1994.tb05903.x 7879636

[25] Anthony WA Recovery from mental illness: the guiding vision of the mental health service system in the 1990s. Psychosoc Rehabil J (1993) 16(4):11–23. 10.1037/h0095655

[26] Leamy M Bird V Le Boutillier C Williams J Slade M Conceptual framework for personal recovery in mental health: systematic review and narrative synthesis. Br J Psychiatry (2011) 199(6):445–52. 10.1192/bjp.bp.110.083733 22130746

[27] Farkas M The vision of recovery today: What it is and what it means for services. World Psychiatry (2007) 6(2):1–7. 18235855 PMC2219905

[28] Farkas M Boevink W Peer delivered services in mental health care in 2018: infancy or adolescence? World Psychiatry (2018) 17(2):222–3. 10.1002/wps.20530 PMC5980530 29856537

[29] Blanch AK Carling PJ Ridgway P Normal housing with specialized supports: a psychiatric rehabilitation approach to living in the community. Rehabil Psychol (1988) 33(1):47–55. 10.1037/h0091686

[30] Doroud N Fossey E Fortune T Place for being, doing, becoming and belonging: A meta-synthesis exploring the role of place in mental health recovery. Heal Place (2018) 52:110–20. 10.1016/j.healthplace.2018.05.008 29885554

[31] Mezzina R Borg M Marin I Sells D Topor A Davidson L From participation to citizenship: how to regain a role, a status, and a life in the process of recovery. Am J Psychiatr Rehabil (2006) 9(1):39–61. 10.1080/15487760500339428

[32] Tew J Ramon S Slade M Bird V Melton J Le Boutillier C Social factors and recovery from mental health difficulties: a review of the evidence. Br J Soc Work. (2012) 42(3):443–60. 10.1093/bjsw/bcr076

[33] Tabol C Drebing C Rosenheck R Studies of "supported" and "supportive" housing: a comprehensive review of model descriptions and measurement. Eval Program Plann. (2010) 33(4):446–56. 10.1016/j.evalprogplan.2009.12.002 20138365

[34] Macpherson R Shepherd G Thyarappa P Supported accommodation for people with severe mental illness: an update. Adv Psychiatr Treat (2012) 18(5):381–91. 10.1192/apt.bp.110.008714

[35] McPherson P Krotofil J Killaspy H What works? Toward a new classification system for mental health supported accommodation services: the simple taxonomy for supported accommodation (STAX-SA). Int J Environ Res Public Health (2018) 15(190):1–17. 10.3390/ijerph15020190 PMC5858263 29364171

[36] The National Academies of Science Permanent supportive housing: evaluating the evidence for improving health outcomes among people experiencing chronic homelessness [Internet]. Washington, DC The National Academies Press (2018). Available from: https://www.nap.edu/read/25133/chapter/210. 30148580

[37] Leff HS Chow CM Pepin R Conley J Allen IE Seaman CA Does one size fit all? What we can and can't learn from a meta-analysis of housing models for persons with mental illness. Psychiatr Serv (2009) 60(4):473–82. 10.1176/ps.2009.60.4.473 19339322

[38] Rog DJ Randolph F A multisite evaluation of supported housing: lessons learned from cross-site collaboration. New Dir Eval (2002) 94:61–72. 10.1002/ev.51

[39] Rog D Randolph FL A multi-site evaluation of supported housing: Lessons learned from a cross-site collaboration. In Herrell JM Straw RB (Eds.), New direction for evaluation: Conducting multiple site evaluations in real world settings. San Francisco: Jossey-Bass (2002).

[40] Tsemberis S Gulcur L Nakae M Housing First, consumer choice, and harm reduction for individuals who are homeless with a dual diagnosis. Am J Public Health (2004) 94(4):651–6. 10.2105/AJPH.94.4.651 PMC1448313 15054020

[41] Aubry T Nelson G Tsemberis S Housing First for people with severe mental illness who are homeless: a review of the research and findings from the at Home-Chez soi demonstration project. Can J Psychiatry (2015) 60(11):467–74. 10.1177/070674371506001102 PMC4679127 26720504

[42] McPherson P Krotofil J Killaspy H Mental health supported accommodation services: a systematic review of mental health and psychosocial outcomes. BMC Psychiatry (2018) 18(1):1–15. 10.1186/s12888-018-1725-8 29764420 PMC5952646

[43] Farkas M Promoting choice: a cornerstone of recovery facilitating services and interventions. Recovery to Practice: newsletter [Internet] Davidson L, editor. Yale School of Medicine. (2012). Available from: https://www.nyaprs.org/e-news-bulletins/2012/farkas-choice-is-a-cornerstone-of-recovery-facilitating-services-interventions.

[44] Sullivan BJ Burke J Single room occupancy housing in New York City: origins and dimensions of a crisis. CUNY Law Rev (2013) 17(1):113–26. 10.31641/clr170104

[45] Szekely B The ultimate in gentrification: the most gentrified ZIP codes in the US. Dowtown LA's 90014 heads the list of fastest-gentrifying ZIPs since the turn of the millennium [Internet]. In: RENTCafé Blog (2018). Available from: https://www.rentcafe.com/blog/rental-market/real-estate-news/top-20-gentrified-zip-codes/.

[46] Fernald M ed. The state of the nations housing Cambridge: MA (2018).

[47] Fernald M ed. The state of the nations housing Cambridge: MA (2016).

[48] Bazelon Center for Mental Health Law A place of my own: how the ADA is creating integrated housing opportunities for people with mental illnesses. Washington, DC: Judge David. Bazelon Center for Mental Health Law (2014).

[49] Bazelon Center for Mental Health Law What fair housing means for people with disabilities [Internet]. In: Washington DC: Judge David L. Bazelon Center for Mental Health Law (2011). Available from: http://www.bazelon.org/wp-content/uploads/2017/01/What-Fair-Housing-Means.pdf.

[50] Hunter J Linden-Retek P Shebaya S Halpert S The rise of tent cities in the United States [Internet]. Metcalf H Tars E Johnson HM. editors. National Law Center on Homelessness & Poverty. Allard K. Lowenstein International Human Rights Clinic, Yale Law School. (2014). p. 152 Available from: https://nlchp.org/wp-content/uploads/2018/10/WelcomeHome_TentCities.pdf.

[51] Henry M Mahathey A Morrill T Robinson A Shivji A Watt R The 2018 annual homeless assessment report to Congress [Internet]. In: Washington, D.C. (2018). Available from: https://files.hudexchange.info/resources/documents/2018-AHAR-Part-1.pdf.

[52] Fazel S Khosla V Doll H Geddes J The prevalence of mental disorders among the homeless in Western countries: systematic review and meta-regression analysis. PloS Med (2008) 5(12):1670–81. 10.1371/journal.pmed.0050225 PMC2592351 19053169

[53] Rog DJ Marshall T Dougherty RH George P Daniels AS Ghose SS Permanent supportive housing: assessing the evidence. Psychiatr Serv (2014) 65(3):287–94. 10.1176/appi.ps.201300261 24343350

[54] Taylor TL Killaspy H Wright C Turton P White S Kallert TW A systematic review of the international published literature relating to quality of institutional care for people with longer term mental health problems. BMC Psychiatry (2009) 9:55. 10.1186/1471-244X-9-55 19735562 PMC2753585

[55] Roberts G Boardman J Becoming a recovery-oriented practitioner. Adv Psychiatr Treat (2014) 20(1):37–47. 10.1192/apt.bp.112.010652

[56] Bird V Leamy M Le-Boutillier C Willliams J Slade M REFOCUS: promoting recovery in mental health services [Internet]. In: section for Recovery, Institute of Psychiatry., King's College (2014). p. 1–34. Available from: researchintorecovery.com/refocus.

[57] Cook JA Shore S Burke-Miller JK Jonikas JA Hamilton M Ruckdeschel B Mental health self-directed care financing: efficacy in improving outcomes and controlling costs for adults with serious mental illness. Psychiatr Serv (2019) 70(3):191–201. 10.1176/appi.ps.201800337 30630401

[58] American Psychological Association African Americans have limited access to mental and behavioral health care. Am Psychol Assoc [Internet] (2018), 1–7. Available from: http://www.apa.org/about/gr/issues/minority/access.aspx.

[59] Ranney ML Gold J The dangers of linking gun violence and mental illness. Time [Internet] (2019), 1–4. Available from: https://time.com/5645747/gun-violence-mental-illness/.

[60] Substance Abuse and Mental Health Services Administration Civil commitment and the mental health care continuum. In: Historical trends and principles for law and practice. Rockville, MD: Office of the Chief Medical Officer, Substance Abuse and Mental Health Services Administration (2019). Available from: https://www.samhsa.gov/sites/default/files/civil-commitment-continuum-of-care.pdf.

